# Complete genome sequence of the gliding freshwater bacterium *Fluviicola taffensis* type strain (RW262^T^)

**DOI:** 10.4056/sigs.2124912

**Published:** 2011-09-23

**Authors:** Tanja Woyke, Olga Chertkov, Alla Lapidus, Matt Nolan, Susan Lucas, Tijana Glavina Del Rio, Hope Tice, Jan-Fang Cheng, Roxanne Tapia, Cliff Han, Lynne Goodwin, Sam Pitluck, Konstantinos Liolios, Ioanna Pagani, Natalia Ivanova, Marcel Huntemann, Konstantinos Mavromatis, Natalia Mikhailova, Amrita Pati, Amy Chen, Krishna Palaniappan, Miriam Land, Loren Hauser, Evelyne-Marie Brambilla, Manfred Rohde, Romano Mwirichia, Johannes Sikorski, Brian J. Tindall, Markus Göker, James Bristow, Jonathan A. Eisen, Victor Markowitz, Philip Hugenholtz, Hans-Peter Klenk, Nikos C. Kyrpides

**Affiliations:** 1 DOE Joint Genome Institute, Walnut Creek, California, USA; 2 Los Alamos National Laboratory, Bioscience Division, Los Alamos, New Mexico, USA; 3 Biological Data Management and Technology Center, Lawrence Berkeley National Laboratory, Berkeley, California, USA; 4 Oak Ridge National Laboratory, Oak Ridge, Tennessee, USA; 5 DSMZ - German Collection of Microorganisms and Cell Cultures GmbH, Braunschweig, Germany; 6 HZI – Helmholtz Centre for Infection Research, Braunschweig, Germany; 7 Jomo Kenyatta University of Agriculture and Technology, Kenya; 8 University of California Davis Genome Center, Davis, California, USA; 9 Australian Centre for Ecogenomics, School of Chemistry and Molecular Biosciences, The University of Queensland, Brisbane, Australia

**Keywords:** strictly aerobic, motile by gliding, Gram-negative, flexirubin-synthesizing, mesophilic, chemoorganotrophic, *Cryomorphaceae*, GEBA

## Abstract

*Fluviicola taffensis* O'Sullivan *et al*. 2005 belongs to the monotypic genus *Fluviicola* within the family *Cryomorphaceae*. The species is of interest because of its isolated phylogenetic location in the genome-sequenced fraction of the tree of life. Strain RW262^T^ forms a monophyletic lineage with uncultivated bacteria represented in freshwater 16S rRNA gene libraries. A similar phylogenetic differentiation occurs between freshwater and marine bacteria in the family *Flavobacteriaceae*, a sister family to *Cryomorphaceae*. Most remarkable is the inability of this freshwater bacterium to grow in the presence of Na^+^ ions. All other genera in the family *Cryomorphaceae* are from marine habitats and have an absolute requirement for Na^+^ ions or natural sea water. *F. taffensis* is the first member of the family *Cryomorphaceae* with a completely sequenced and publicly available genome. The 4,633,577 bp long genome with its 4,082 protein-coding and 49 RNA genes is a part of the *** G****enomic* *** E****ncyclopedia of* *** B****acteria and* *** A****rchaea * project.

## Introduction

Strain RW262^T^ (= DSM 16823 = NCIMB 13979) is the type strain of the species *Fluviicola taffensis*, which is the type species of the monotypic genus *Fluviicola* [1], affiliated with the family *Cryomorphaceae* [2]. The genus name is derived from the Latin words *fluvius*, meaning 'river' and *-cola* meaning 'inhabitant, dweller', yielding the Neo-Latin word *Fluviicola*, the *river dweller* [1,3]. The species epithet is derived from the Neo-Latin word *taffensi*s, referring to the place where the type strain has been isolated, the river Taff (Wales, UK) [1,3]. The family *Cryomorphaceae* belongs to the class *Flavobacteria* which contains many species that probably play an integral role for the flow of carbon and energy in the marine environment [4]. *Flavobacteria* are the major decomposers of high-molecular-mass organic matter in sea water [5]. Phylogenetically the family *Cryomorphaceae* is located between the families *Flavobacteriaceae* and *Bacteroidaceae* [2] and currently comprises the genera *Brumimicrobium*, *Cryomorpha* and *Crocinitomix* [2], *Owenweeksia* [6], *Wandonia* [7], *Fluviicola* [1] and *Lishizhenia* [8]. The family *Cryomorphaceae* exhibits the greatest degree of phenotypic similarity to the family *Flavobacteriaceae* [9] and includes species with a mostly rod-like to filamentous morphology; cells are usually non-motile or move by gliding and often contain carotenoid pigments [1,2]. All members of the *Cryomorphaceae* are strictly aerobic or facultatively anaerobic (fermentative) with a chemoheterotrophic metabolism [1,2] and often have complex growth requirements for sea water salts, organic compounds as sole nitrogen sources, yeast extract and vitamins for growth [2]. To date no further isolates of *F. taffensis* have been reported. Here we present a summary classification and a set of features for *F. taffensis* RW262^T^, together with the description of the complete genomic sequencing and annotation.

## Classification and features

A representative genomic 16S rRNA sequence of *F. taffensis* RW262^T^ was compared using NCBI BLAST [10] under default settings (e.g., considering only the high-scoring segment pairs (HSPs) from the best 250 hits) with the most recent release of the Greengenes database [11] and the relative frequencies of taxa and keywords (reduced to their stem [12]) were determined, weighted by BLAST scores. The most frequently occurring genera were *Brumimicrobium* (62.9%) and *Fluviicola* (37.1%) (3 hits in total). Among all other species, the one yielding the highest score was *'Brumimicrobium mesophilum'* (DQ660382), which corresponded to an identity of 92.1% and an HSP coverage of 58.0%. (Note that the Greengenes database uses the INSDC (= EMBL/NCBI/DDBJ) annotation, which is not an authoritative source for nomenclature or classification.) The most frequently occurring keywords within the labels of all environmental samples which yielded hits were 'lake' (9.1%), 'tin' (3.4%), 'microbi' (2.5%), 'depth' (2.0%) and 'tract' (1.7%) (247 hits in total). The most frequently occurring keywords within those labels of environmental samples which yielded hits of a higher score than the highest scoring species were 'lake' (9.2%), 'tin' (3.8%), 'microbi' (2.3%), 'depth' (2.0%) and 'tract' (1.8%) (169 hits in total). The most frequent keyword 'lake' may reflect the freshwater origin of strain RW262^T^, whereas the keywords 'tin' and 'depth' may allude to some until now unrecognized ecological features of *F. taffensis.*

**Figure 1:**
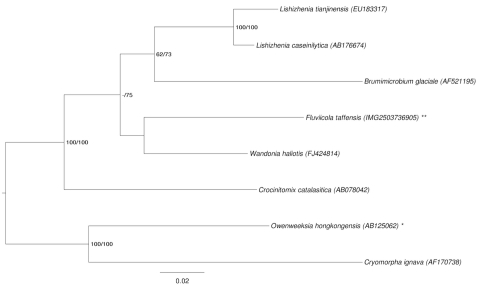
Phylogenetic tree highlighting the position of *F. taffensis* relative to the type strains of the other species within the family *Cryomorphaceae*. The tree was inferred from 1,429 aligned characters [13,14] of the 16S rRNA gene sequence under the maximum likelihood (ML) criterion [15]. Rooting was done initially using the midpoint method [16] and then checked for its agreement with the current classification (Table 1). The branches are scaled in terms of the expected number of substitutions per site. Numbers adjacent to the branches are support values from 300 ML bootstrap replicates [17] (left) and from 1,000 maximum parsimony bootstrap replicates [18] (right) if larger than 60%. Lineages with type strain genome sequencing projects registered in GOLD [19] are labeled with one asterisk, those also listed as 'Complete and Published' (as well as the target genome) with two asterisks.

Figure 1 shows the phylogenetic neighborhood of *F. taffensis* in a 16S rRNA based tree. The sequences of the two identical 16S rRNA gene copies in the genome differ by two nucleotides from the previously published 16S rRNA sequence (AF493694), which contains one ambiguous base call.

**Table 1:** Classification and general features of *F. taffensis* RW262^T^ according to the MIGS recommendations [20] and the NamesforLife database [21].

MIGS ID	Property	Term	Evidence code
	Current classification	Domain *Bacteria*	TAS [22]
		Phylum *Bacteroidetes*	TAS [23]
		Class “*Flavobacteria*”	TAS [24]
		Order “*Flavobacteriales*”	TAS [25]
		Family “*Cryomorphaceae*”	TAS [2]
		Genus *Fluviicola*	TAS [1]
		Species *Flaviicola taffensis*	TAS [1]
		Type strain RW262	TAS [1]
	Gram stain	negative	TAS [1]
	Cell shape	rod-shaped	TAS [1]
	Motility	by gliding	TAS [1]
	Sporulation	none	TAS [1]
	Temperature range	4°C-25°C	TAS [1]
	Optimum temperature	20°C	TAS [1]
	Salinity	obligate 0%	TAS [1]
MIGS-22	Oxygen requirement	strict aerobe	TAS [1]
	Carbon source	probably amino acids; unable to use carbohydrates	NAS
	Energy metabolism	chemoorganotroph	TAS [1]
MIGS-6	Habitat	fresh water	TAS [1]
MIGS-15	Biotic relationship	free-living	NAS
MIGS-14	Pathogenicity	none	NAS
	Biosafety level	1	TAS [26]
	Isolation	fresh river water	TAS [1]
MIGS-4	Geographic location	River Taff near Cardiff, UK	TAS [1]
MIGS-5	Sample collection time	January 2000	TAS [1]
MIGS-4.1	Latitude	51.85	TAS [1]
MIGS-4.2	Longitude	-2.32	TAS [1]
MIGS-4.3	Depth	not reported	
MIGS-4.4	Altitude	sea level	NAS

Evidence codes - TAS: Traceable Author Statement (i.e., a direct report exists in the literature); NAS: Non-traceable Author Statement (i.e., not directly observed for the living, isolated sample, but based on a generally accepted property for the species, or anecdotal evidence). These evidence codes are from of the Gene Ontology project [27].

**Figure 2:**
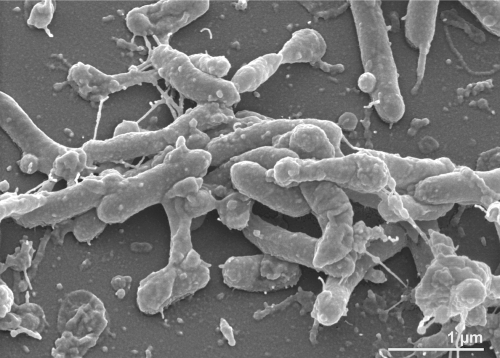
Scanning electron micrograph of *F. taffensis* RW262^T^

Strain RW262^T^ is strictly aerobic, Gram-negative, motile by gliding and flexirubin-pigmented [1]. Cells are flexible rods with rounded ends (Figure 2), 0.4-0.5 µm in diameter and 1.5-5.7 µm in length, with rare longer filaments of up to 51 µm in length [1]. Growth occurs at 4ºC and 20ºC, but not in the presence of Na^+^ ions [1]. Growth of strain RW262^T^ at 4 ºC is only weak, so that *F. taffensis* should not be considered to be psychrotolerant like the other members of the family [1,2]. Strain RW262^T^ is capable of DNA hydrolysis [1], is catalase positive but oxidase negative, able to catalyze the hydrolysis of arginine, aesculin or starch, whereas it weakly hydrolyzes gelatine [1]. It is negative for nitrate and nitrite reduction; indole production; β-galactosidase, urease and xylanase activity; hydrolysis of agar, arginine, aesculin and starch; and acid production from carbohydrates [1]. The strain is not able to utilize glucose, arabinose, mannose, mannitol, *N*-acetylglucosamine, maltose, gluconate, caprate, adipate, malate, citrate or phenyl acetate [1]. However, within the genome are several genes for utilization of complex organic carbon compounds. The strain is resistant to chloramphenicol (10 µg), streptomycin (10 µg), and kanamycin (30 µg) but susceptible to penicillin G (10 units), ampicillin (10 µg), rifampicin (5 µg) and tetracycline (10 µg) [1].

### Chemotaxonomy

The predominant cellular acid of strain RW262^T^ was the branched-chain saturated fatty acid *iso*-C_15:0_ (44.2%) [1]. Unsaturated branched-chain fatty acids, straight-chain saturated and mono-unsaturated fatty acids occur only in lower amounts: C_14:0_ (3.2%), C_15:0_ (7.5%), C_16:0_ (3.0%), *iso*-C_15:1 ω10c_ (11.8%), *iso*-C_16:1 ω12c_ (4.9%). Lipopolysaccharide hydroxy fatty acids constitute 20.4% of the total cellular fatty acids, mainly composed of *iso*-C_17:0 3-OH_ (12.3%), iso-C_15:0 3-OH_ (4.2%) and iso-C_15:0 2-OH_ (3.5%) [1].

## Genome sequencing and annotation

### Genome project history

This organism was selected for sequencing on the basis of its phylogenetic position [28], and is part of the *** G****enomic* *** E****ncyclopedia of* *** B****acteria and* *** A****rchaea * project [29]. The genome project is deposited in the Genome On Line Database [19] and the complete genome sequence is deposited in GenBank. Sequencing, finishing and annotation were performed by the DOE Joint Genome Institute (JGI). A summary of the project information is shown in Table 2.

**Table 2:** Genome sequencing project information

**MIGS ID**	**Property**	**Term**
MIGS-31	Finishing quality	Finished
MIGS-28	Libraries used	Tree genomic libraries: one 454 pyrosequence standard library, one 454 PE library (11 kb insert size), one Illumina library
MIGS-29	Sequencing platforms	Illumina GAii, 454 GS FLX Titanium
MIGS-31.2	Sequencing coverage	351.0 × Illumina; 23.0 × pyrosequence
MIGS-30	Assemblers	Newbler version 2.3, Velvet, phrap version SPS – 4.24
MIGS-32	Gene calling method	Prodigal 1.4, GenePRIMP
	INSDC ID	CP002542
	Genbank Date of Release	April 1, 2011
	GOLD ID	Gc01706
	NCBI project ID	47603
	Database: IMG-GEBA	2503707007
MIGS-13	Source material identifier	DSM 16823
	Project relevance	Tree of Life, GEBA

### Growth conditions and DNA isolation

*F. taffensis* RW262^T^, DSM 16823, was grown in DSMZ medium 948 (Oxoid nutrient medium) [30] at 28°C. DNA was isolated from 0.5-1 g of cell paste using JetFlex Genomic DNA Purification kit (GENOMED 600100) following the standard protocol as recommended by the manufacturer, but with additional 20 µl proteinase K incubation (one hour) at 58° for improved cell lysis. DNA is available through the DNA Bank Network [31].

### Genome sequencing and assembly

The genome was sequenced using a combination of Illumina and 454 sequencing platforms. All general aspects of library construction and sequencing can be found at the JGI website [32]. Pyrosequencing reads were assembled using the Newbler assembler (Roche). The initial Newbler assembly consisting of 51 contigs in one scaffold was converted into a phrap [33] assembly by making fake reads from the consensus, to collect the read pairs in the 454 paired end library. Illumina GAii sequencing data (801.4 Mb) was assembled with Velvet [34] and the consensus sequences were shredded into 1.5 kb overlapped fake reads and assembled together with the 454 data. The 454 draft assembly was based on 164.9 Mb 454 draft data and all of the 454 paired end data. Newbler parameters are -consed -a 50 -l 350 -g -m -ml 20. The Phred/Phrap/Consed software package [33] was used for sequence assembly and quality assessment in the subsequent finishing process. After the shotgun stage, reads were assembled with parallel phrap (High Performance Software, LLC). Possible mis-assemblies were corrected with gapResolution [32], Dupfinisher [35], or sequencing clones bridging PCR fragments with subcloning. Gaps between contigs were closed by editing in Consed, by PCR and by Bubble PCR primer walks (J.-F. Chang, unpublished). A total of 161 additional reactions and shatter libraries were necessary to close gaps and to raise the quality of the finished sequence. Illumina reads were also used to correct potential base errors and increase consensus quality using a software Polisher developed at JGI [36]. The error rate of the completed genome sequence was less than 1 in 100,000. Together, the combination of the Illumina and 454 sequencing platforms provided 374.0 × coverage of the genome. The final assembly contained 232,904 pyrosequence and 44,902,395 Illumina reads.

### Genome annotation

Genes were identified using Prodigal [37] as part of the Oak Ridge National Laboratory genome annotation pipeline, followed by a round of manual curation using the JGI GenePRIMP pipeline [38]. The predicted CDSs were translated and used to search the National Center for Biotechnology Information (NCBI) non-redundant database, UniProt, TIGR-Fam, Pfam, PRIAM, KEGG, COG, and InterPro databases. Additional gene prediction analysis and functional annotation was performed within the Integrated Microbial Genomes - Expert Review (IMG-ER) platform [39].

## Genome properties

**Figure 3:**
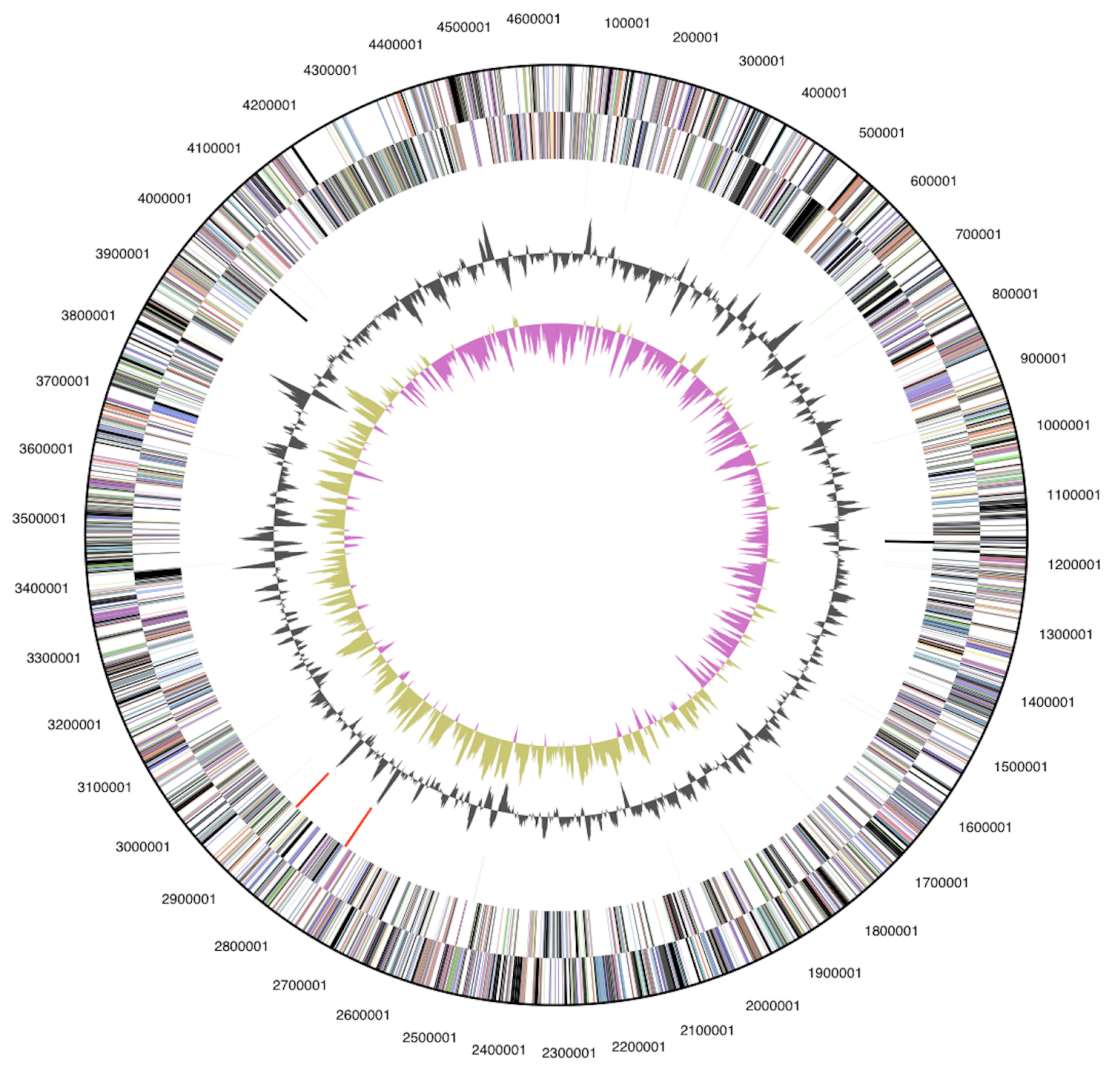
Graphical circular map of the genome. From outside to the center: Genes on forward strand (color by COG categories), Genes on reverse strand (color by COG categories), RNA genes (tRNAs green, rRNAs red, other RNAs black), GC content, GC skew.

The genome consists of a 4,633,577 bp long chromosome with a G+C content of 36.5% (Table 3 and Figure 3). Of the 4,131 genes predicted, 4,082 were protein-coding genes, and 49 RNAs; 49 pseudogenes were also identified. The majority of the protein-coding genes (55.0%) were assigned a putative function while the remaining ones were annotated as hypothetical proteins. The distribution of genes into COGs functional categories is presented in Table 4.

**Table 3:** Genome Statistics

**Attribute**	**Value**	**% of Total**
Genome size (bp)	4,633,577	100.00%
DNA coding region (bp)	4,192,830	90.49%
DNA G+C content (bp)	1,691,009	36.49%
Number of replicons	1	
Extrachromosomal elements	0	
Total genes	4,131	100.00%
RNA genes	49	1.19%
rRNA operons	2	
Protein-coding genes	4,082	98.81%
Pseudo genes	49	1.19%
Genes with function prediction	2,271	54.97%
Genes in paralog clusters	532	12.88%
Genes assigned to COGs	2,169	52.51%
Genes assigned Pfam domains	2,420	58.58%
Genes with signal peptides	1,331	32.22%
Genes with transmembrane helices	911	22.05%
CRISPR repeats	1	

**Table 4:** Number of genes associated with the general COG functional categories

**Code**	**value**	**%age**	**Description**
J	168	6.0	Translation, ribosomal structure and biogenesis
A	0	0.0	RNA processing and modification
K	212	8.8	Transcription
L	137	5.7	Replication, recombination and repair
B	1	0.0	Chromatin structure and dynamics
D	22	0.9	Cell cycle control, cell division, chromosome partitioning
Y	0	0.0	Nuclear structure
V	57	2.4	Defense mechanisms
T	183	7.6	Signal transduction mechanisms
M	222	9.2	Cell wall/membrane/envelope biogenesis
N	8	0.3	Cell motility
Z	0	0.0	Cytoskeleton
W	0	0.0	Extracellular structures
U	42	1.8	Intracellular trafficking, secretion, and vesicular transport
O	106	4.4	Posttranslational modification, protein turnover, chaperones
C	117	4.9	Energy production and conversion
G	76	3.2	Carbohydrate transport and metabolism
E	136	5.7	Amino acid transport and metabolism
F	63	2.6	Nucleotide transport and metabolism
H	114	4.7	Coenzyme transport and metabolism
I	101	4.2	Lipid transport and metabolism
P	116	4.8	Inorganic ion transport and metabolism
Q	45	1.9	Secondary metabolites biosynthesis, transport and catabolism
R	286	11.9	General function prediction only
S	194	8.1	Function unknown
-	1,962	47.5	Not in COGs
